# Guiding Undergraduates Through the Process of First Authorship

**DOI:** 10.3389/fpsyg.2019.00857

**Published:** 2019-04-18

**Authors:** Traci A. Giuliano

**Affiliations:** Department of Psychology, Southwestern University, Georgetown, TX, United States

**Keywords:** undergraduate research, undergraduate publication, publishing, first authorship, faculty-student collaboration

## Introduction

Dozens of excellent papers have recently been written that describe best practices for publishing journal articles with undergraduates (see “Engaging Undergraduates in Publishable Research: Best Practices,” *Frontiers in Psychology*); for the most part, these involve students as co-authors in general rather than as lead authors. In this paper, I specifically focus on how to guide undergraduates through the process of first authorship. After describing potential barriers, I discuss issues of authorship contribution before outlining several successful strategies I've developed during my 24 years of collaborating with undergraduates. Although mentoring students to be first authors can be challenging, the rewards can also be immense—for both the students and the faculty mentors who are up to the challenge.

## The Undergraduate First Author: a Unicorn?

A literature search revealed not a single article on the topic of undergraduates publishing as first author. Without any data, it's hard to know for certain how common it is for undergraduates to publish as first authors, but informal discussions with psychology colleagues around the world who collaborate with undergraduates (and examinations of faculty vitae) suggest that it is far less common than undergraduates publishing as non-lead authors.

## Barriers (Real or Perceived) to Undergraduate First Authorship

Because it is rare to see undergraduate first authors, many faculty are likely unaware that at least some undergraduates can—with proper training, encouragement, and careful mentoring—be capable of serving as first authors on papers in refereed journals. Even if faculty members are made aware of this fact (as I hope to accomplish with this article), other barriers exist. For example, many faculty work under a reward system in which publications (and first author publications in particular) determine tenure, promotion, pay, likelihood of securing grants, and job security (e.g., Costa and Gatz, 1992; Fine and Kurdek, 1993; Wilcox, 1998). The primary tradeoff is that the time it takes to mentor undergraduates through first authorship is generally much longer than the time it would take for the faculty member to be the lead author. The great experience provided to the student (see Matthews and Rosa, 2018), therefore, can come at the cost of decreased productivity (e.g., fewer publications overall, fewer first author publications, publications in lower-tier journals), which could be problematic for faculty at institutions that don't highly value faculty-undergraduate research. Finally, recent trends in psychological science, such as the difficulty of publishing single-study papers in some subfields and the “open science” movement calling for large sample sizes, pre-registration, and replication (see Chambers, 2017; Nelson et al., 2018) can seem like roadblocks to publishing with undergraduates. Fortunately, faculty from diverse subfields have come up with creative solutions involving high-quality replications (e.g., McKelvie and Standing, 2018; Wagge et al., 2019), preregistered projects (e.g., Strand and Brown, 2019), large-scale single-experiment class projects designed for publication (e.g., LoSchiavo, 2018; Mickley Steinmetz and Reid, 2019), and multi-study projects involving student coauthors across years (e.g., Grysman and Lodi-Smith, 2019; Holmes and Roberts, 2019).

## Authorship Contribution and Order of Authorship

Much has been written about the ethics of assigning authorship credit in the sciences and social sciences (see Maurer, 2017, for a review), and attempts have been made to fairly determine authorship order by (a) surveying past authors about their experiences (e.g., Wagner et al., 1994; Sandler and Russell, 2005; Moore and Griffin, 2006; Geelhoed et al., 2007), (b) assessing reactions to hypothetical authorship scenarios (e.g., Costa and Gatz, 1992; Bartle et al., 2000; Apgar and Congress, 2005), (c) proposing step-by-step decision-making models (Fine and Kurdek, 1993; Foster and Ray, 2012; Maurer, 2017), and (d) outlining quantitative systems that assign weighted points to tasks associated with publishing (e.g., Winston, 1985; Kosslyn, 2015). The consensus seems to be that writing the manuscript is either the most important factor in determining first authorship (e.g., Winston, 1985; Bartle et al., 2000; Apgar and Congress, 2005) or at least tied with idea origination as the most important factor (Wagner et al., 1994; Kosslyn, 2015). The “authorship determination scorecard” on the American Psychological Association's website (https://www.apa.org/science/leadership/students/authorship-paper.aspx), for example, allots 170 of 1,040 points (16%) for idea generation/refinement; 110 points (11%) for design/measures; 160 points (15%) for statistical analysis, and 600 points (58%) for writing/revision.

Given the clear importance of writing as a factor in first authorship, and because students' contributions to idea generation, design, and analysis are often similar to those of their collaborators up to this point, I always require students to take responsibility for the manuscript drafts and revisions (with my feedback and editing help) to earn their first authorship. I am typically second author (consistent with the “order of contribution” norm in social psychology) because I play a significant role in the publication process, but less than the first author. The remaining student authors tend to be less involved (consistent with Geelhoed et al., 2007) because of lack of time or interest, or geographical distance. Nonetheless, all authors are always asked to read and approve the final manuscript before submission.

## Paths to Undergraduate First Authorship

My mentor, the late Dan Wegner (a social psychologist who ended his career at Harvard but started at a small liberal arts university doing research with undergraduates) advised me as I began my career at an undergraduate-only institution that “the best undergraduates are often better than graduate students” because they are “not only very bright, but often are more intrinsically motivated—if you hold them to high standards, they will meet or exceed them, and you can publish great work with them.” I followed his advice, and indeed have published the vast majority of my papers with undergraduates as co-authors, and especially as first authors: Of my 33 post-graduate school publications, 29 papers involve a total of 68 undergraduate co-authors, and 24 of the 29 are first-authored by undergraduates^1^.

In my experience, there have been three primary paths to undergraduate first authorship, each representing approximately one-third of my publications with students. First, during our one-semester research methods course with a lab (capped at 12 students), sophomores and sometimes juniors complete two original projects and manuscript write-ups, and conducting high quality, original projects is a big factor (see LoSchiavo, 2018); about 10% of my class projects lead to publication. Second, each faculty member has a capstone course in which they work with 5 to 6 seniors (or sometimes juniors) for two consecutive semesters; about 90% of my capstone projects lead to publication^2^. Third, I occasionally accept projects for individual honors theses or independent studies (independent research outside of capstone is rare in our department, perhaps one senior every several years) if I think they are publishable; about 90% of these projects lead to publication.

## Best Practices

Here are some of the strategies I've developed over the years to successfully mentor students to first authorship:

1. **Provide good writing instruction throughout the curriculum**. It is crucial to teach good writing skills throughout the curriculum (Soysa et al., 2013) so that the largest number of students possible has a strong background and the potential capacity to be first author. (My university has 1,400 students, and we graduate 25–30 psychology majors annually, so with 4–5 faculty members striving to publish with students each year, this step is crucial). Our department places a strong emphasis on students learning APA style as well as proper grammar (see Giuliano, 2019), and all instructors provide copious feedback on student drafts. Although group writing is popular elsewhere (e.g., small groups of students who write APA-style papers together on their research methods project), instructors in our department require individual writing (as well as peer review) in both research methods and capstone courses so that every student improves and gets the maximum amoun of practice.
2. **Select the most “first-author-ready” students**. I've found that it is important to select students with certain characteristics—those who not only have the strongest writing skills, but who are hardworking, independent, intellectually curious, and intrinsically motivated^3^. The process starts when I read a paper (e.g., a research methods final paper, a senior capstone paper, or an honors thesis) that has good results, that is “close enough” that I can envision grooming it into a publishable paper, and that has been written by a student with the characteristics described above.
3. **Explain what authorship entails**. At that point, I ask the student if she or he would like to first author a publication under my supervision (Virtually every invitee will have already first-authored a conference presentation with me, so I know that we are a “good fit” and that they know exactly what to expect when working with me.). As recommended by Foster and Ray (2012), I explain which contributions determine first authorship: I tell them they have already earned authorship by making significant contributions in the idea, design, and analysis stages, as have their student collaborators, so they will earn first authorship by being responsible for writing the manuscript, with plenty of feedback and supervision from me. To provide “informed consent” about this decision (Fine and Kurdek, 1993), I outline clear expectations (i.e., that they can expect to write 10–15 drafts or more over a period of several months, that this will be a much higher standard of writing than they have ever done in the past, and that at times this process could get frustrating and tedious) and let them know that they are free to accept or decline without any adverse consequences (about 95% of students accept). I also tell them that first authorship is not guaranteed and that authorship order may need to be revised if contributions change (Only once or twice in 24 years has first authorship changed; my students have generally been excellent at following through with their commitments.).
4. **Get them ready to write**. Once students agree to be first author, the next step is to provide them with exemplar articles (I use past publications from my own students). I then set an initial calendar of deadlines (e.g., when their drafts are due to me, when my feedback is due to them); I usually draft this first and then allow students to make modifications according to their schedule. Finally, I have students research and take notes on potential target journals (we then discuss the pros and cons together and decide where to send the paper once finished).
5. **Find time to write**. Finding time to write can be tricky, because students are often either busy with other courses or have moved on to jobs or graduate school. Summers are usually optimal for both students and me. For research methods class projects, I usually suggest writing during the summer after the course is over (setting the final deadline before the new semester starts). If students are in town, we meet in person occasionally but generally trade drafts over email and have in-person or by-phone meetings when necessary. Writing with students who have graduated is often more difficult because those with jobs are busy working during the day and no longer in “academic mode,” so I find that it takes more patience and encouragement to get them back into the writing. If they are in graduate school, they are already immersed in research, which is helpful, but projects with their graduate advisor compete for their attention. Students who have graduated are also more likely to be out of town, which is only a problem if in-person meetings (e.g., to re-analyze data) are necessary, although online meeting applications (e.g., Facetime, Skype) work fine. Ultimately, it may take some creativity to find the time and space for writing, as in “writing weekends” (see Scherman, under review), but in the end, it is worth it.

## Conclusion

Publishing with students is truly my favorite part of being a professor—the thrill I get upon seeing a student's name in print (especially in the lead position) is often greater than the thrill I get from seeing my own name. As others have argued (e.g., Malachowski, 2012; Maurer, 2017), when working with students, it is best to treat them as equals and true partners in the collaboration process, with high levels of autonomy and a strong focus on student learning. In doing so, the rewards—for both students and faculty alike—are incredibly worthwhile.

## Author Contributions

The author confirms being the sole contributor of this work and has approved it for publication.

### Conflict of Interest Statement

The author declares that the research was conducted in the absence of any commercial or financial relationships that could be construed as a potential conflict of interest.

## Footnotes

^1^Four are in the *Psi Chi Journal of Undergraduate Research*; the remainder are in professional, peer-reviewed journals.

^2^It should be noted that our department recently switched from an informal system in which either faculty or students approached the other about the possibility of research collaboration to a more formal capstone assignment process in which all students (during their required research methods course) complete a written application describing their interest in conducting a research-based capstone and rank their preference for faculty labs. This process not only improved transparency, but also provided more equitable information, access, and opportunity for all students, who are assigned to labs by fit and interest. Recent articles have addressed both the benefits of increasing diversity and inclusion in undergraduate research and publication (e.g., Peifer, 2019) and specific strategies for doing so (e.g., Ahmad et al., under review; Chan, 2019; Scisco et al., 2019) and are highly recommended.

^3^Approximately half of my first authors went on to Ph.D. programs in psychology; the other half went to law school, medical school, master's programs, or did not seek a graduate degree.

## Undergraduate^*^ First-Author Publications

^*^Butterworth, S. E., Giuliano, T. A., ^*^White, J. R., ^*^Cantu, L., & ^*^Fraser, K. C. (In Press). Is he flirting with me? How sender gender influences emoji interpretation. *Frontiers in Psychology*.

^*^Matthews, S. J., Giuliano, T. A., ^*^Rosa, M. N., ^*^Thomas, K. H., ^*^Swift, B. A., ^*^Ahearn, N. D., ^*^Garcia, A. G., ^*^Smith, S. R., ^*^Niblett, C. M., & ^*^Mills, M. M. (2018). The battle against bedroom boredom: Development and validation of a brief measure of sexual novelty in relationships. *Canadian Journal of Human Sexuality, 27*, 277-287.

^*^Matthews, S. J., Giuliano, T. A., ^*^Thomas, K. H., ^*^Straup, M. L., & ^*^Martinez, M. A. (2018). Not cool, dude: Perceptions of solicited vs. unsolicited sext messages from men and women. *Computers in Human Behavior, 88*, 1-4. 10.1016/j.chb.2018.06.14

^*^Matthews, S. J., Giuliano, T. A., ^*^Rosa, M. N., ^*^Thomas, K. H., & ^*^Swift, B. A. (2018). Sexual Novelty Scale. Handbook of Sexuality-Related Measures. Thousand Oaks, CA: Sage Publications.

^*^Hutzler, K. T., Giuliano, T. A, ^*^Herselman, J. R., & ^*^Johnson, S. M. (2015). Three's a crowd: Public awareness and (mis)perceptions of polyamory. *Psychology & Sexuality, 7*, 69-87. 10.1080/19419899.2015.1004102

^*^Johnson, S. M., Giuliano, T. A, ^*^Herselman, J. R., & ^*^Hutzler, K. T. (2015). Development of a brief measure of attitudes towards polyamory. *Psychology & Sexuality, 6*, 325-339. 10.1080/19419899.2014.1001774

^*^Blomquist, B.A., & Giuliano, T. A. (2012). “Do you love me, too?” Perceptions of Responses to ‘I love you.” *North American Journal of Psychology, 14*, 407-418.

^*^Gomillion, S. C., & Giuliano, T. A. (2011). The influence of media role models on gay, lesbian, and bisexual identity. *Journal of Homosexuality, 58*, 330-354.

^*^Howell, J., ^*^ & Giuliano, T. A. (2011). The effects of expletive use and team gender on perceptions of coaching effectiveness. *Journal of Sport Behavior, 34*, 69-81.

^*^Howell, J., ^*^Egan, P., ^*^Ackley, B., & Giuliano, T. A. (2011). The reverse double standard in perceptions of student-teacher sexual relationships: The role of gender, initiation, and power. *Journal of Social Psychology, 151*(2), 180-200.

^*^Egan, P., & Giuliano, T. A. (2009). Unaccommodating attitudes: Perceptions of students with learning disabilities as a function of accommodation use and test performance. *North American Journal of Psychology, 11*, 487-500.

^*^Osborne, R. L, ^*^Ackley B. D, & Giuliano, T. A., (2008). The “skinny” on coffee drinkers: Gender differences in healthy beverage choice. *Psi Chi Journal of Undergraduate Research, 13(4)*, 159-163.

^*^Riggs, A. L., & Giuliano, T. A. (2007). Running in the family or swimming in the gene pool: The role of family history and genetic risk in individuals' illness perceptions. *Journal of Health Psychology, 12*, 883-894.

^*^Stanzer, M., Guarraci, F., Giuliano, T. A., & Sims, A. (2007). Paramedic or EMT-basic partner? Study evaluates preferred partner types & the effect of partners on work-related stress levels. *Journal of Emergency Medical Services 32:* 72-74.

^*^Knight, J. L., & Giuliano, T. A. (2003). Blood, sweat, and jeers: The impact of the media's heterosexist portrayals on perceptions of male and female athletes. *Journal of Sport Behavior, 26*, 272-284.

^*^Wilke, K. M., ^*^Turner, K. L., & Giuliano, T. A. (2003). Smoke screens: Cross-cultural effectiveness of anti-smoking messages. *North American Journal of Psychology, 5*, 431-442.

^*^Dodd E. H., Giuliano, T. A., ^*^Boutell, J. M., & ^*^Moran, B. E. (2001). Respected or rejected: Perceptions of women who confront sexist remarks. *Sex Roles*, 45, 567-577.

^*^Knight, J. L., & Giuliano, T. A. (2001). She's a “looker;” he's a Laker: The consequences of gender-stereotypical portrayals of male and female athletes by the print media. *Sex Roles, 45*, 217-229.

^*^Knight, J. L., Giuliano, T. A., & ^*^Sanchez-Ross, M. G. (2001). Famous or infamous? The influence of celebrity status and race on perceptions of responsibility for rape. *Basic and Applied Social Psychology, 23*, 183-190.

^*^Dickson, A., Giuliano, T. A., ^*^Morris, J. C., & ^*^Cass, K. L. (2001). Eminem versus Charley Pride: Race, stereotypes, and perceptions of rap and country music performers. *Psi Chi Journal of Undergraduate Research, 6*, 175-179.

^*^Kirkendall, K. D., ^*^Dixon, D. P., Giuliano, T. A., & ^*^Raney, A. E. (2001). The bold and the beautiful: The effect of physical attractiveness and extraversion on desirability. *Psi Chi Journal of Undergraduate Research, 6*, 180-186.

^*^Cohorn, C. A., & Giuliano, T. A. (1999). Predictors of adjustment and institutional attachment in first-year college students. *Psi Chi Journal of Undergraduate Research, 4*, 47-56.

^*^Cox, C. B., & Giuliano, T. A. (1999). Constructing obstacles vs. making excuses: Examining perceivers' reactions to behavioral and self-reported self-handicapping. *Journal of Social Behavior and Personality,14*, 419-432.

^*^Fiala, S. E., Giuliano, T. A., ^*^Remlinger, N. M., & ^*^Braithwaite, L. C. (1999). Lending a helping hand: The effects of sex stereotypes and gender on likelihood of helping. *Journal of Applied Social Psychology, 29*, 2164-2176.
